# Ataxic Severity Is Positively Correlated With Fatigue in Spinocerebellar Ataxia Type 3 Patients

**DOI:** 10.3389/fneur.2020.00266

**Published:** 2020-04-22

**Authors:** Jin-Shan Yang, Hao-Ling Xu, Ping-Ping Chen, Arif Sikandar, Mei-Zhen Qian, Hui-Xia Lin, Min-Ting Lin, Wan-Jin Chen, Ning Wang, Hua Wu, Shi-Rui Gan

**Affiliations:** Department of Neurology and Institute of Neurology, First Affiliated Hospital, Fujian Medical University, Fuzhou, China

**Keywords:** fatigue, spinocerebellar ataxia type 3, neurodegeneration, clinical manifestation, ataxic severity

## Abstract

**Background:** Spinocerebellar ataxia type 3 (SCA3) is an inherited form of ataxia that leads to progressive neurodegeneration. Fatigue is a common non-motor symptom in SCA3 and other neurodegenerative diseases, such as Parkinson's disease (PD) and amyotrophic lateral sclerosis (ALS). Although risk factors to fatigue in these diseases have been thoroughly studied, whether or not fatigue can affect clinical phenotypes has yet to be investigated.

**Methods:** Ninety-one molecularly confirmed SCA3 patients and 85 age- and sex-matched controls were recruited for this study. The level of fatigue was measured using the 14-item Fatigue Scale (FS-14), and the risk factors to fatigue and how fatigue correlates with clinical phenotypes were studied using multivariable linear regression models.

**Results:** We found that the severity was significantly higher in the SCA3 group than in the control group (9.30 ± 3.04% vs. 3.94 ± 2.66, *P* = 0.000). Daytime somnolence (β = 0.209, *P* = 0.002), severity of ataxia (β = 0.081, *P* = 0.006), and poor sleep quality (β = 0.187, *P* = 0.037) were found to have a positive relationship with fatigue. Although fatigue had no relationship with age at onset or ataxic progression, we found that it did have a positive relationship with the severity of ataxia (β = 7.009, *P* = 0.014).

**Conclusions:** The high level of fatigue and the impact of fatigue on the clinical manifestation of SCA3 patients suggest that fatigue plays a large role in the pathogenesis of SCA3, thus demonstrating the need for intervention and treatment options in this patient cohort.

## Introduction

Spinocerebellar ataxia type 3 (SCA3), which was initially known as Machado–Joseph disease (MJD), is the most common subtype of spinocerebellar ataxias (SCAs) worldwide and one of autosomal dominant neurodegenerative disorders. SCA3 is caused by the dynamic mutation involving CAG repeat expansion in exon 10 of the *ATXN3* gene on chromosome 14q32.1, which results in a pathologically glutamine tract within the encoded ataxin-3 (1). It is clinically characterized by progressive cerebellar ataxia, dysphagia, oculomotor disturbances, and extrapyramidal signs. In addition to the cardinal motor manifestations, SCA3 may also exhibit several non-motor clinical manifestations such as sleep behavior disorders, cognitive deficits, olfactory dysfunction, psychiatric symptoms, nutritional problems, autonomic nervous function disturbances, and fatigue (2, 3).

Fatigue, which is clinically defined as difficulty in the initiation of or sustainment of voluntary mental and physical activities (4), is a subjective experience and common in neurodegenerative disorders such as Parkinson's disease (PD) and amyotrophic lateral sclerosis (ALS) (5–12). As a common symptom, fatigue was found to have great impact on the quality of life in PD (13–16). Fatigue is also a pronounced non-motor symptom in SCAs (17–19), especially in SCA3 patients, with a high prevalence around 52.9–63.5% (3, 20, 21). The significant predictors of fatigue, such as length of expanded CAG repeats, excessive daytime somnolence, and mood disorders such as anxiety and major depression have been explored (3, 19, 20, 22, 23). Given that the frequency of fatigue is so high and severity worsens over time with disease progression (4), the question is raised as to whether or not it could affect the clinical symptoms seen in neurodegenerative disorders, which have never been investigated.

For this study, we recruited 91 molecularly confirmed SCA3 patients and 85 age- and sex-matched controls to study the frequency of fatigue and the risk factors to fatigue in our subjects. And then, correlation between fatigue and clinical manifestations such as age at onset (AAO), severity, and progression of ataxia was further investigated.

## Subjects and Methods

### Study Subjects

Ninety-one molecular-confirmed SCA3 patients and 85 healthy individuals were recruited between October 2014 and January 2018. In order to be eligible to participate, patients had to meet our inclusion criteria as follows: (1) the presence of ataxia, (2) definite genetic diagnosis of SCA3, (3) willingness to participate, and (4) age of 20 years and older. Exclusion criteria were the following: (1) the presence of homozygotes for SCA3, (2) met exclusion criteria of SCA3 by a previous genetic test, and (3) had concomitant disorder(s) that affect the International Cooperative Ataxia Rating Scale (ICARS), which is the ataxia measurement used in this study. The control group was matched for age, gender, and environmental characteristics including mostly spouses, caregivers of SCA3/MJD patients, and unrelated healthy person. Relatives at risk were excluded from the control group.

This study was approved by the ethics committee of the First Affiliated Hospital of Fujian Medical University, and written informed consents were signed by all participants prior to study commencement.

### Genotype and Phenotype Analysis

Genomic DNA was extracted from peripheral blood using QIAamp DNA Blood Mini kit (Qiagen, Hilden, Germany) following standard procedures. The numbers of subjects' CAG repeats were determined by polymerase chain reaction (PCR) amplification combined with Sanger sequencing as reported in our previous studies (24, 25). Based on the CAG repeat numbers, alleles of *ATXN3* could be divided into normal alleles (CAG repeat numbers ≤44) and expanded alleles (CAG repeat numbers ≥50) (25).

Each patient participated in a face-to-face interview with ataxia specialists (SRG, HLX, and JSY) to collect demographic data and neurological features. The severity of ataxia was assessed using semiquantitative International Cooperative Ataxia Rating Scale (ICARS), which involves four subscales of postural and stance disorders, limb ataxia, dysarthria, and oculomotor disorders, with a sum score ranging from 0 (absence of ataxia) to 100 (severe ataxia) (26). AAO was defined as the time when the patient or close relatives/caregivers could recall the first appearance of any symptoms related to SCA3. Disease duration was defined as the time span between AAO and the age at first visit. Progression degree of ataxia was measured by a patient's ICARS score divided by duration.

In order to rate the severity of fatigue, the 14-item Fatigue Scale (FS-14) was used. The FS-14, which is a 14-item questionnaire, reflects the severity of two different kinds of fatigue (physical fatigue and mental fatigue), and a higher score suggests severer fatigue (27). Since the study subjects were all Chinese, we adopted a Chinese version of FS-14, which was proved to exhibit optimal sensitivity and specificity in recognizing fatigue among the Chinese-speaking population (28) and was widely used for valuing the severity of fatigue (29–34). To value the severity of daytime somnolence, sleep disturbances, and depression, which were reported to related to the severity of fatigue (19, 20, 22), patients were asked to complete the Chinese version of questionnaires of the Epworth Sleepiness Scale (ESS), the Pittsburgh Sleep Quality Index (PSQI), and the Beck Depression Inventory (BDI). The ESS assesses sleep propensity in eight different daily situations with a total score ranging from 0 (no excessive daytime sleepiness) to 24 (severe excessive daytime sleepiness). Patients were classified as having excess daytime somnolence (EDS) if the ESS score was at least 10 (35). The PSQI measures sleep quality and disturbances using seven components, resulting in a total score ranging from 0 to 21. Poor sleep quality was indicated if the PSQI score was >5 (36). BDI is a 21-question, self-rating tool that measures symptom of depression. Standard cutoff scores range from 0 to 63, with a higher score indicating severe depression. For the purposes of this study, a BDI score of ≥19 was defined as clinically relevant depression (37).

### Statistical Analyses

Variables with normal distribution valued by Kolmogorov–Smirnov test were expressed as the mean ± SD and evaluated by Student's independent sample *t*-test. Variables with skewed distribution were expressed as mean ± SD and median and evaluated by one-way analysis of variance (ANOVA). Gender difference between SCA3 patients and controls was analyzed with chi-square test.

To determine possible factors relative to fatigue, we used a multivariable logistic regression model. The FS-14 score was dichotomized into two groups based on the value of median. The FS-14 score (binary) was the dependent variable. The AAO, gender (binary), disease duration, the length of normal and expanded CAG repeats, ICARS, ESS, BDI, and PSQI scores were considered independent variables.

To analyze whether fatigue was correlated with clinical phenotypes (e.g., AAO, severity of ataxia, and progression of ataxia) of SCA3 patients, a multivariable linear regression model was performed. To determine the influence of fatigue on AAO, it was set as the dependent variable. Scores from the FS-14 (binary), ESS, BDI, and PSQI, along with gender (binary), CAG repeat numbers of normal alleles, and expanded alleles, were regarded as the independent variables. In the model of analyzing the relationship between fatigue and severity of ataxia, ICARS score was considered the dependent variable. Scores from the FS-14 (binary), ESS, BDI, and PSQI along with gender (binary), AAO, disease duration, and CAG repeat numbers of normal alleles and expanded alleles were considered independent variables. To explore the relationship between fatigue and progression of ataxia, we used the progression degree of ataxia as the dependent variable and scores of FS-14 (binary), ESS, BDI, and PSQI along with gender (binary), AAO, and the length of normal and expanded CAG repeats as the independent variables.

To detect whether the data were suitable for logistic or linear regression model, the goodness of fit was tested by calculating *R*^2^. The closer the value of *R*^2^ to 1, the better goodness of fit the equation had.

All statistical analyses were performed using SPSS version 27.0 (SPSS Inc., Chicago, IL, USA). The results were considered statistically significant at *P* < 0.05.

## Results

The demographic features of 91 SCA3 patients and 85 controls are summarized in Table 1. Of the 91 subjects with SCA3, 53 (58.24%) were men and 38 (41.76%) were women. The mean AAO was 34.24 ± 9.48 years, and average disease duration was 8.20 ± 5.09 years. The average numbers of CAG repeat in normal and expanded allele were 21.20 ± 9.20 and 74.59 ± 3.73, respectively. The control group was composed of 85 healthy normal person. The gender and age distribution between SCA3 patients and controls showed no significant differences (*P* = 0.761 for gender; *P* = 0.217 for age).

**Table 1:** The demographic characteristics of study subjects.

	**Variable distribution**	**Controls**	**SCA3 patients**	***P*-value**
Sample size, *N* (%)	NA	85	91	NA
Age of first visit (year)	Normal	43.91 ± 11.99	42.43 ± 10.82	0.217^c^
Gender (M/F)		47/38	53/38	0.761^a^
Disease duration (year)	NA	NA	8.20 ± 5.09 Median = 7.00	NA
Age at onset (year)	NA	NA	34.24 ± 9.48 Median = 34	NA
Length of expanded CAG repeats, *N*	NA	NA	74.59 ± 3.73 Median = 74.00	NA
Length of normal CAG repeats, *N*	Skewed	NA	21.20 ± 9.20 Median = 14.00	NA
ICARS score	NA	NA	28.79 ± 15.56 Median = 28.00	NA
FS-14 score	Skewed	3.94 ± 2.66 Median = 3.00	9.30 ± 3.04 Median = 10.00	**0.000**^b^
ESS score	Skewed	5.94 ± 3.51 Median = 6.00	7.68 ± 5.24 Median = 6.00	**0.003**^b^
BDI score	Skewed	8.91 ± 6.88 Median = 8.00	23.33 ± 14.08 Median = 24.00	**0.000**^b^
PSQI score	Skewed	4.41 ± 2.01 Median = 4.00	7.77 ± 4.59 Median = 7.00	**0.000**^b^
Progression degree of ataxia	NA	NA	4.71 ± 3.66 Median = 3.78	NA

*BDI, Beck Depression Inventory; ESS, Epworth Sleepiness Scale; F, female; FS-14; 14-Item Fatigue Scale; ICARS, International Cooperative Ataxia Rating Scale; M, male; N, number; NA, non-application; PSQI, Pittsburgh Sleep Quality Index; SCA, spinocerebellar ataxia*.

*Values represent mean ± standard deviation or number, and for variables with non-normal distribution, the median is reported as well. Bold value showed significance*.

a *Chi-square test*.

b *Mann–Whitney U-test*.

c *Two-independent samples t-test*.

The mean FS-14 score was significantly higher in the SCA3 group than in the control group (9.30 ± 3.04, median = 10 vs. 3.94 ± 2.66, median = 3, *P* = 0.000). Otherwise, ESS, PSQI, and BDI scores were also higher in SCA3 patients than in the control subjects (7.68 ± 5.24 vs. 5.94 ± 3.51, *P* = 0.003; 7.77 ± 4.59 vs. 4.41 ± 2.01, P = 0.000; 23.33 ± 14.08 vs. 8.91 ± 6.88, *P* = 0.000, respectively) (Table 1). The proportion of SCA3 patients with EDS (ESS score ≥10), poor sleep quality (PSQI score ≥5), as well as depression (BDI score ≥19) were also higher than controls (37.36 vs. 11.76%, *P* = 0.000, 72.53 vs. 48.24%, *P* = 0.001, 59.34 vs. 4.71%, *P* = 0.000, respectively).

To investigate the factors associated with fatigue, a multivariable logistic regression model was performed. After adjusting for AAO, gender, disease duration, length of normal CAG repeats, and scores of BDI and PSQI, we found that daytime somnolence (β = 0.209, *P* = 0.002), severity of ataxia (β = 0.081, *P* = 0.006), and the poor sleep quality (β = 0.187, *P* = 0.037) were positively correlated with fatigue (Table 2). The *R*^2^ for the regression model of fatigue was 0.509.

**Table 2:** Testing risk factors relevant to fatigue.

	**Coefficient estimate**	**Standard error**	***P*-value**
Gender^a^	−0.134	0.603	0.824
AAO	0.034	0.044	0.436
Expanded CAG repeats	0.202	0.123	0.101
Normal CAG repeats	0.055	0.038	0.145
Disease duration	−0.087	0.072	0.229
ICARS	0.081	0.030	**0.006**
ESS	0.209	0.068	**0.002**
BDI	−0.003	0.024	0.892
PSQI	0.187	0.090	**0.037**

*AAO, age at onset; ESS, Epworth Sleepiness Scale; BDI, Beck Depression Inventory; PSQI, Pittsburgh Sleep Quality*.

*Bold value showed significance*.

a *Female vs. male*.

The multivariable linear regression model was further utilized to evaluate correlative factors of SCA3 clinical phenotypes. After adjusting for AAO, gender, disease duration, length of normal and expanded CAG repeats, and scores of ESS, BDI, and PSQI, we found that fatigue was positively correlated with severity of ataxia (β = 7.009, *P* = 0.014). There was no significant correlation between fatigue and AAO (β = 2.265, *P* = 0.165) or fatigue and disease progression (β = 0.066, *P* = 0.945) (Table 3). The *R*^2^ for the regression model of ataxia severity was 0.551.

**Table 3:** The influences of fatigue on clinical phenotypes.

	**Coefficient estimate**	**Standard error**	***P*-value**
**The influence of fatigue on AAO**			
Gender^a^	3.889	1.392	**0.006**
Expanded CAG repeats	−1.860	0.188	**0.000**
Normal CAG repeats	−0.195	0.077	**0.013**
FS-14^b^	2.265	1.619	0.165
ESS	0.006	0.140	0.964
BDI	−0.002	0.057	0.974
PSQI	0.039	0.183	0.831
**The influence of fatigue on ataxic severity**			
Gender^a^	4.039	2.545	0.116
AAO	0.219	0.188	0.246
Expanded CAG repeats	0.334	0.474	0.484
Normal CAG repeats	0.019	0.140	0.890
Disease duration	1.311	0.256	**0.000**
FS-14^b^	7.009	2.788	**0.014**
ESS	−0.361	0.242	0.140
BDI	0.166	0.101	0.106
PSQI	0.751	0.314	**0.019**
**The influence of fatigue on disease progression**^c^			
Gender^a^	−0.679	0.849	0.426
AAO	0.041	0.064	0.522
Expanded CAG repeats	0.124	0.162	0.445
Normal CAG repeats	0.057	0.047	0.224
FS-14^b^	−0.066	0.955	0.945
ESS	0.014	0.082	0.864
BDI	−0.010	0.033	0.770
PSQI	0.063	0.107	0.522

*AAO, age at onset; BDI, Beck Depression Inventory; ESS, Epworth Sleepiness Scale; FS-14, 14-Item Fatigue Scale; PSQI, Pittsburgh Sleep Quality Index*.

a *Female vs. male*.

b *The group above median vs. the group below median*.

c *Disease progression: ICARS scores divided by disease duration (in years)*.

*Bold value showed significance*.

## Discussion

In the present study, we found that SCA3 patients have significant higher level of fatigue, compared to the normal controls. We also found that daytime somnolence and ataxic severity are risk factors associated with fatigue. While daytime somnolence, the length of expanded CAG repeats, anxiety, and depression have been reported to be associated with fatigue (3, 19, 20, 22, 23), it was the first time that ataxic severity was found to be associated with fatigue. The significant correlation between fatigue and disease severity was also revealed in a PD cohort (8). The positive relationship between fatigue and clinical features was initially investigated in SCA3 in the present study. We found that although fatigue had no correlations with AAO and ataxic progression, it did have a significant positive relationship with the severity of ataxia.

Our findings suggest that there is a bidirectional relationship between ataxia and fatigue in SCA3 patients. However, the physiological mechanisms to this close connection remains unknown and is typically considered multifactorial. Normally, fatigue can be divided into either central fatigue, which is caused by lesions in pathways associated with arousal and attention, reticular and limbic systems, thalamus, and the basal ganglia or peripheral fatigue, which is caused by neuromuscular junction disorders or metabolic myopathy (4). Previous studies have demonstrated that dopaminergic dysfunction of the basal ganglia is one of the potential mechanisms for central fatigue in PD patients (38, 39). Similarly, abnormalities of the dopamine transporter were observed in SCA3 patients (40). Additionally, one study showed that there was widespread microstructural white matter damage in the thalamus, bilateral cerebral white matter, and the frontal and temporal lobes in SCA3 patients (24). Therefore, we speculated that abnormalities in the dopamine transporter and white matter of cerebra may contribute to the central fatigue observed in SCA3 patients. It is tangible that the peripheral fatigue noted in SCA3 patients may be due to peripheral neuropathy, since symptoms such as muscle cramps, weakness, numbness, muscle wasting, neuropathic pain, and disturbance of skeletal muscle energy metabolism are common in SCA3 patients (41, 42).

The influence of fatigue on ataxic severity highlights the need to prevent fatigue in SCA3. Until now, a treatment for fatigue in SCA3 patients has not yet been reported. However, standardized treatment for fatigue in PD patients does exist, inclusive of a balanced medication regime targeting movement disorder (e.g., dopaminergic agents) and depression (e.g., psychostimulants and antidepressants) combined with a rehabilitation program (e.g., cognitive behavior therapy and graded exercise) (5). Furthermore, research has demonstrated that pharmacological (modafinil) and non-pharmacological (respiratory exercise and repetitive transcranial magnetic stimulation) management may be possible treatment options for fatigue in ALS patients (43). These reports indicate that the appropriate combination of pharmacological and non-pharmacological treatments could be promising treatment options for alleviating fatigue and the severity of ataxia in SCA3 patients.

## Conclusions

In summary, the high frequency of fatigue and its impact on the clinical manifestation in SCA3 patients suggests that it plays a large role in the pathogenesis of SCA3 and necessitates the development of an intervention and treatment plan in SCA3 patients.

## Data Availability Statement

All datasets generated for this study are included in the article/supplementary material.

## Ethics Statement

The studies involving human participants were reviewed and approved by First Affiliated Hospital of Fujian Medical University. The patients/participants provided their written informed consent to participate in this study.

## Author Contributions

J-SY and H-LX were in charge of study concept and design, statistical analysis and interpretation, and writing of the manuscript. HW and S-RG were in charge of study concept and design and critical revision of the manuscript for important intellectual content. W-JC and NW designed and supervised the research. P-PC, AS, M-ZQ, H-XL, and M-TL collected and analyzed data.

## Conflict of Interest

The authors declare that the research was conducted in the absence of any commercial or financial relationships that could be construed as a potential conflict of interest.
